# Physical properties and biological/odontogenic effects of an experimentally developed fast-setting α-tricalcium phosphate-based pulp capping material

**DOI:** 10.1186/1472-6831-14-87

**Published:** 2014-07-11

**Authors:** Jun-Bong Lee, Su-Jung Park, Hyun-Ha Kim, Young-Sun Kwon, Kwang-Won Lee, Kyung-San Min

**Affiliations:** 1 Department of Conservative Dentistry, School of Dentistry and Institute of Oral Bioscience, Chonbuk National University, 567 Baekje-daero, Jeonju, Korea; 2 Research Institute of Clinical Medicine of Chonbuk National University-Biomedical Research Institute of Chonbuk National University Hospital, 20 Geonji-ro, Jeonju, Korea; 3 Department of Conservative Dentistry, School of Dentistry, Wonkwang University, 460 Iksan-daero, Iksan, Korea

**Keywords:** Calcium phosphate, Fast-setting, Mineral trioxide aggregate, Odontogenic, Pulp capping, Tertiary dentin

## Abstract

**Background:**

Recently, fast-setting α-tricalcium-phosphate (TCP) cement was developed for use in the pulp capping process. The aim of this study was to investigate the physical properties and biological effects of α-TCP cement in comparison with mineral trioxide aggregate (MTA).

**Methods:**

We measured the setting time, pH values, compressive strength, and solubility of the two materials. We evaluated biocompatibility on the basis of cell morphology and a viability test using human dental pulp cells (hDPCs). Chemical composition of each material was analyzed by energy dispersive x-ray spectroscopic (EDS) analysis. The expression of odontogenic-related genes was evaluated by Western blotting and immunofluorescence. The calcified nodule formation was measured by Alizarin red staining. We performed the pulp capping procedure on rat teeth for histological investigation. The data were analyzed by an independent *t-*test for physical properties, one-way ANOVA for biological effects, and the Mann-Whitney *U* test for tertiary dentin formation. A *P* value of less than 0.05 was considered statistically significant for all tests.

**Results:**

The setting time, pH values, and compressive strength of α-TCP was lower than that of MTA (*P* < 0.05); however, the solubility of α-TCP was higher than that of MTA (*P* < 0.05). The resultant cell viability observed with the two materials was similar (*P* > 0.05). Scanning electron microscopy (SEM) revealed that cells attached to both materials were flat and had cytoplasmic extensions. The expression of odontogenic-related markers and mineralized nodule formation were higher in the two experimental groups compared to the control group (*P* < 0.05). Continuous tertiary dentin was formed underneath the capping materials in all samples of the tested groups.

**Conclusions:**

Our study demonstrated that the α-TCP exhibited biocompatibility and odontogenicity comparable to MTA, whereas it had a quicker setting time.

## Background

Calcium phosphate (CP) cements have been used for repairing bone defects [1,2]. They are reportedly good candidates for osseous augmentation by virtue of their biocompatibility, moldability and osteoconductivity [3,4]. Thus, there have been many dental trials investigating the use of these materials on periodontal defects [5-8]. In addition, many studies have shown that CP cements stimulate pulp and can induce the formation of reparative dentin [9-14]. Researchers also showed the superior physical properties of calcium phosphate cements compared to calcium hydroxide, a traditional pulp capping material introduced in the 1930s. However, CP cements were observed to have limitations, including long setting times and low compressive strength, when used alone as pulp-capping agents. Meanwhile, mineral trioxide aggregate (MTA) was introduced to the endodontic field in the 1990s [15]. It was demonstrated that MTA possesses favorable physical and biological properties, and displays excellent potential in endodontic applications, such as direct pulp capping [16-18]. From that time, most studies regarding pulp capping materials have focused on MTA, and CP cements have been out of the spotlight. However, MTA also has some drawbacks including long setting time [19], initial looseness [20], and poor handling characteristics [21].

Recently, a fast-setting α-tricalcium phosphate (TCP)-based cement (Mediclus, Cheongju, Korea) was developed experimentally to overcome the disadvantage of conventional CP cements. According to manufacturer, it was developed not only for endodontic use, including pulp therapy, root-end filling, and perforation repair, but also for periodontal/surgical use, such as in osseous regeneration, which has been considered a primary application of CP cements. In other words, in addition to setting time, α-TCP cement may be more advantageous in variety of clinical applications compared to MTA if the recommended properties are met. However, to our knowledge, there has been no prior study to evaluate this fast-setting α-TCP-based cement as a pulp capping material. Therefore, in the present study, we showed the possibility of the α-TCP cement for use in pulp capping applications. The aim of the study was to investigate its setting time, compressive strength, solubility, biocompatibility, and odontogenic effect in comparison with MTA on the basis of *in vitro* and *in vivo* pulp capping experimental models. Our two null hypotheses were as follows: (i) There is no difference between MTA and α-TCP regarding physical properties and (ii) there is no difference between these two materials with respect to biological/odontogenic effects.

## Methods

### Measurement of setting time

We mixed MTA (ProRoot; Dentsply, Tulsa, OK, USA) and α-TCP according to the manufacturers’ instructions. Then, the samples (n = 10) were tested just before their anticipated setting time and at 30-second intervals until they were fully set. A Gilmore apparatus was used with a stainless steel indenter and 1/4-pound indentation force for the initial setting time measurement; a 1-pound indentation force was used for the final setting time. We applied the apparatus at a right angle to the surface of the sample for 5 sec. The setting time was defined as the time at which the indenter failed to leave a definite mark on the sample surface.

### Measurement of pH

Specimens (1-mm thickness and 5-mm diameter) of MTA and α-TCP were prepared and allowed to set for 1 day (n = 3). After setting, one tablet was inserted into 10 mL of deionized water. Then, the resultant pH value was measured using a pH meter (Orion 3 Star; Thermo Scientific, Singapore). The apparatus was previously calibrated with pH 7.0 and pH 4.0 solutions. Between each measurement the electrode was washed with ultrapure water and blot-dried.

### Solubility

After mixing in accordance with the manufacturers’ instructions, samples of each material were placed in a paraffin wax mold 1.5-mm-thick and 20 mm in diameter (n = 5). Each sample was weighed using an analytical balance and the weight was recorded as W_1_. The samples were then immersed in glass flasks containing 10 mL distilled water and the flasks were sealed. Samples were removed at 7 days, dried with absorbent paper, and placed in a desiccator. The samples were dried to a constant weight (±0.001 g), which was recorded as W_2_. The solubility (S) was calculated using the following formula: S = (W_1_ – W_2_) / W_1_ × 100.

### Measurement of compressive strength

We determined the compressive strength of the test materials by using the method recommended by ISO 3107:2004 (n = 10). Each material was mixed and placed in a split stainless steel mold (4-mm in diameter and 6-mm in height) within 2 min after the start of mixing. The complete assembly was transferred to a cabinet maintained at 37°C for 6 hours.

The specimens were removed from the molds and checked visually for any air-voids or chipped edges. All defective specimens were discarded, and 10 acceptable samples were prepared for each test material at each time interval. The specimens were immersed in distilled water for 1, 7, 14, and 28 days and maintained at 37°C.

We then measured the compressive strength of each sample by using a universal testing machine at a crosshead speed of 1.0 mm/min. The maximum load required to fracture each specimen was determined. The compressive strength was calculated in megapascals (MPa) with the following formula: C = 4P/D^2^, where P is the applied force (N) and D is the diameter (mm) of the specimen. The compressive strength of all specimens was recorded in MPa.

### Primary culture of hDPCs

Human dental pulp tissues obtained from sectioned teeth were removed aseptically, rinsed with phosphate buffered saline solution (PBS; HyClone Laboratories, Logan, UT, USA), and placed in a 60-mm dish (Nunc, Roskilde, Denmark). Then, we minced the dental pulp tissues with a blade into small fragments and cultured in minimal essential medium-α (MEM-α; HyClone Laboratories) containing 10% fetal bovine serum (FBS; Invitrogen, Carlsbad, CA, USA) along with 100 U/mL penicillin and 100 U/mL streptomycin (Invitrogen). Cultures were maintained at 37°C in a humidified atmosphere of 5% CO_2_ and 95% air. Cell cultures between the third to fifth passages were used in this study. All experimental procedures were approved by the Institutional Review Board (IRB#: CUH 2013-01-015) of the Chonbuk National University Hospital (Jeonju, Korea).

### Preparation of material extracts

We mixed the tested materials according to the manufacturers’ instructions. The mixed cement was placed into a paraffin wax mold (1-mm thickness and 5-mm diameter), and the cement was stored in an incubator at 100% relative humidity and 37°C for 1 day of hydration. The cements were then sterilized under ultraviolet light for 1 h. One tablet of each cement was stored in 10 mL of MEM-α containing 10% FBS for 3 days.

### Cell viability test

We seeded cells in 24-well culture plates (SPL Lifesciences, Pocheon, Korea) at a density of 2 × 10^4^ cells per well and pre-incubated in growth medium for 24 h. Then, the cells were treated with the prepared extracts (experimental groups) or medium-only (control group). After exposure to the material extracts for 1, 2, 3, 7, and 14 days, cell viability was examined using the 3-(4,5-dimethylthiazol-2-yl)-2,5-diphenyltetrazolium bromide (MTT) assay. Briefly, 200 μL of MTT solution (0.5 mg/ml in PBS) was added to each well, and the wells were incubated for 2 hours. Subsequently, 200 μL of dimethyl sulfoxide (DMSO; Amresco, Solon, OH, USA) was added to each well. The plates were then shaken until the MTT crystals had dissolved, and the solution in each well was transferred to a 96-well tissue culture plate. Reduced MTT was then measured spectrophotometrically at 540 nm in a dual-beam microtiter plate reader (SPECTROstar Nano; BMG Labtech, Ortenberg, Germany).

### Cell morphological observation using SEM

Under aseptic conditions, we condensed the materials into 1 × 5-mm round wax molds. The materials were allowed to set for 24 h in a humidified incubator at 37°C. Then, the disks were placed at the bottom of 24-well tissue culture plates (SPL Lifesciences). Cells were seeded at 1 × 10^5^ cells per well on the prepared materials. After a 72-h incubation period, the dishes were fixed with 2.5% glutaraldehyde (Sigma-Aldrich, St. Louis, MO, USA) for 2 h. Samples were then dehydrated in increasing concentrations of ethanol (70%, 80%, 90%, 95%, and 100%) for 20 min at each concentration and immersed in n-butyl alcohol (Junsei Chemical Co., Tokyo, Japan) for 20 min. SEM was performed using an SN-3000 system (Hitachi, Tokyo, Japan) operated at 10 kV.

### Energy dispersive x-ray spectroscopic (EDS) analysis

We executed EDS analysis using an Apollo-X detector (EDAX, Mahwah, NJ, USA), which was attached to a scanning electron microscope, for chemical element analysis of the surface of MTA and α-TCP. The high magnification of × 10,000 was selected to discern the chemical compositions of specific crystal types within a sample. Via this process, a spectrum was obtained, and elements could be identified. Semi-quantitative, standard-less analyses of these spectra were performed to derive the atomic percent concentrations of constituent elements.

### Western blotting

We seeded hDPCs (3 × 10^5^) in MEM-α containing 10% FBS in 100-mm culture plates and incubated for 24 h. The medium was then switched to the extract medium. After exposure to the extract medium for 3 days, cell lysates were prepared by solubilizing the cells with protein lysis buffer (Pro-prep; iNtRON Biotechnology, Seongnam, Korea) for 10 min on ice. The cell lysates were centrifuged at 13,000 rpm for 10 min, and protein concentrations were determined with Bradford reagent (Bio-Rad Laboratories, Hercules, CA, USA). Samples containing equal amounts of protein were separated by sodium dodecyl sulfate-polyacrylamide gel electrophoresis (SDS-PAGE) and transferred to nitrocellulose transfer membranes (Protran; Whatman, Dassel, Germany). The membranes were blocked with 5% skim milk in TBST at room temperature for 30 min and incubated overnight at 4°C with primary antibodies against dentin sialophosphoprotein (DSPP; Santa Cruz Biotechnology, Santa Cruz, CA, USA), dentin matrix protein 1 (DMP1; Santa Cruz Biotechnology), osteonectin (ON; Santa Cruz Biotechnology), or glyceraldehyde-3-phosphate dehydrogenase (GAPDH; Thermo Scientific, Rockford, IL, USA), followed by incubation with HRP-conjugated secondary antibodies. Antibody-bound proteins were detected using the ECL Western Blotting Luminol reagent (Santa Cruz Biotechnology). The intensity of DSPP, DMP1, and ON protein expression after normalization with GAPDH was quantified using an image analysis program (Image J; National Institutes of Health, Bethesda, MD, USA).

### Alizarin red S staining for mineralized nodule formation

The cells were placed in a 24-well plate at a density of 1 × 10^5^ cells per well and cultured for 24 h. Then, the medium was switched to material extract for the duration of the experiment. After exposure to the extract medium for 14 days, mineralization was assessed by staining with Alizarin red S (Sigma-Aldrich). In brief, 40 mmol/L of Alizarin red S was prepared in distilled water, adjusted to a pH of 4.2 with ammonium hydroxide, and then applied to the cells for 10 min at room temperature with gentle agitation. After being washed with de-ionized water, the stained cell culture plate was moved to a scanner, and the stained image was acquired. For quantitative evaluation, the sample was reacted with 10% cetylpyridinium chloride solution (pH 7.0; Sigma-Aldrich) at room temperature for 15 min to dissolve the stain, and then absorbance was measured at a wavelength of 540 nm with a standard solution.

### Immunofluorescence analysis

Glass coverslips were sterilized by dipping them in 90% ethanol, and then carefully drying them over a flame. Then, a coverslip was placed in each well of a sterile 6-well tissue culture plate. Cell suspensions containing 1 × 10^4^ cells/mL were added to each coverslip. After incubating the cells for 24 h, the medium was switched to material extract. After exposure to the extract medium for 7 days, cells were fixed in 4% paraformaldehyde for 20 min at room temperature. Then, they were incubated in 0.1% Triton X-100 in PBS for 15 min. After blocking with 10% goat serum for 1 h at room temperature, cells were incubated for 2 h with monoclonal mouse anti-DSPP (Santa Cruz Biotechnology), anti-DMP1 (Santa Cruz Biotechnology), or anti-ON (Santa Cruz Biotechnology) (1:100) in 10% goat serum. Then, the cells were incubated with fluorophore-conjugated secondary antibodies (anti-mouse-FITC) for 2 h at room temperature. Coverslips were mounted onto slides using mounting solution. Fluorescent images were obtained using a fluorescence microscope (Carl Zeiss, Jena, Germany).

### Surgical procedure

Twenty healthy upper first molars from 10 eight-week old male Wistar rats were used for this study. Occlusal class I cavities were prepared, and then pinpoint pulpal exposure was made on the occlusal surface of the upper first molar using a #1/8 round carbide bur at high speed under water cooling. Then, the teeth were randomly divided into two test groups, one in which MTA (n = 6) was used to cap the teeth, and the other in which α-TCP (n = 6) was used. The materials were mixed according to the manufacturers’ recommendations, and then applied to the exposure site. The cap was covered with a thin layer of light-cured glass ionomer cement (Fuji II LC; GC, Tokyo, Japan). Four teeth in the control group were capped only with glass ionomer cement. After four weeks, the rats were sacrificed by transcardial perfusion with 4% paraformaldehyde in PBS. The experimental procedures were approved by the Institutional Animal Care and Use Committees (IACUC#: WKU13-14) of Wonkwang University (Iksan, Korea).

### Histological examination

The maxillary segments were dissected carefully, immersed in 4% paraformaldehyde, and kept at 4°C for 24 h. After decalcification using 18% ethylene diamine tetraacetic acid (EDTA; Yakuri Pure Chemical, Osaka, Japan) solution, the specimens were embedded in paraffin, sectioned (5-μm thickness), and stained with hematoxylin-eosin. Tertiary dentin formation was scored according to the criteria used in a previously published study with slight modification (Table 1) [22].

**Table 1:** Scores used for dentinal bridge formation

**Score**	**Characterization**
1	Complete
2	Little communication of the capping material with dental pulp
3	Only lateral deposition of hard tissue on the walls of the cavity
4	Absence of hard tissue bridge

### Statistical analysis

The data for physical properties were analyzed by an independent samples *t-*test to compare the two materials. Statistical analysis was performed by one-way ANOVA followed by a multiple-comparison Tukey’s test for the cell viability test, western blotting, and Alizarin red staining. The Mann-Whitney *U* test was used to evaluate tertiary dentin formation. A *P* value of less than 0.05 was considered statistically significant for all tests.

## Results

### Setting time

The initial setting time of the MTA was 68 min (±5 min), and the final setting time was 284 min (±10 min). The initial setting time of the α-TCP was 4 min (±30 s), and the final setting time was 6 min (±30 s). The setting time of the α-TCP was significantly shorter than that of the MTA (*P* < 0.05).

### Measurement of pH values, solubility, and compressive strength

**Figure 1:**
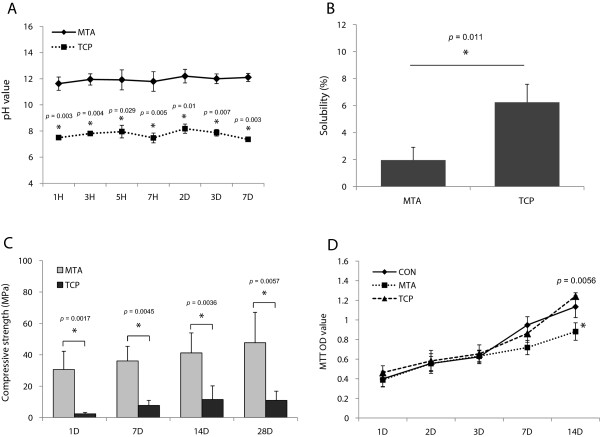
**The physical properties and cell viability of the tested materials.** The pH values **(A)**, solubility **(B)**, and compressive strength **(C)** of MTA and TCP. Note that the setting time, pH values, and compressive strength of α-TCP was lower than that of MTA whereas the solubility of α-TCP was higher than that of MTA. **(D)** Effects of MTA and TCP on cell viability measured by MTT assay. The cell viability of α-TCP-treated samples was higher than those of MTA at day 14. *Significant difference between each group; *P* < 0.05.

The pH values of α-TCP showed mild alkalinity, whereas MTA showed high alkalinity around 11–12 (Figure 1A). The pH of the solution never exceeded 8.2 in the presence of α-TCP throughout the experimental period. Consequently, the pH values of MTA were significantly higher than those of α-TCP (*P* < 0.05). The solubility of α-TCP was higher than that of MTA after 7 days (*P* < 0.05) (Figure 1B). As shown in Figure 1C, the compressive strength of MTA was significantly higher than that of α-TCP at all time intervals (*P* < 0.05). Furthermore, the compressive strength of both MTA and α-TCP increased with time.

### Cell viability test

To evaluate cell viability in the presence of the material extracts, a MTT assay was performed. As shown in Figure 1D, MTA and α-TCP exhibited similar cell viability until day 7 (*P* > 0.05). However, the cell viability of α-TCP-exposed samples was higher than those of MTA at day 14 (*P* < 0.05).

### Cell morphologic analysis

**Figure 2:**
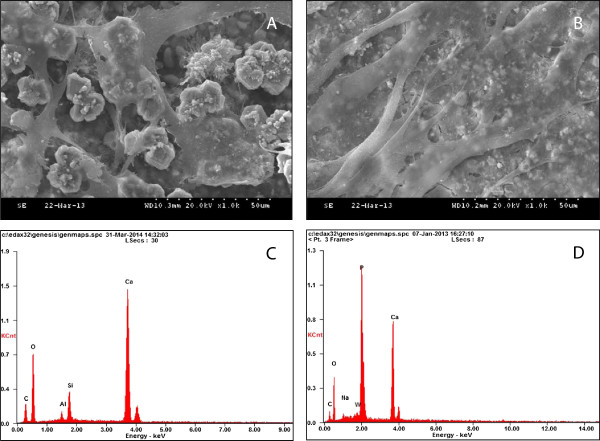
**Investigation of cell morphology and chemical composition of the materials.** SEM observation of cells incubated for 3 days on **(A)** MTA (×1000) and **(B)** TCP (×1000). Both groups showed flattened cells in close proximity to one another, and these were seen to be spreading across the substrate. EDS analysis of the samples: **(C)** MTA and **(D)** TCP.

The cell growth and morphology on each material was evaluated by using SEM observation. As shown in Figure 2A and B, well-spread and flattened hDPCs were observed in contact with the surfaces of MTA and α-TCP.

### Energy dispersive x-ray spectroscopic (EDS) analysis

To investigate the chemical composition of the materials, EDS analysis was performed. The EDS spectra for elemental identification showed that MTA contained calcium (Ca) and silicon (Si) as the major elemental constituents whereas TCP did Ca and phosphate (P) (Figure 2C and D).

### Expression of odontogenic-related markers

**Figure 3:**
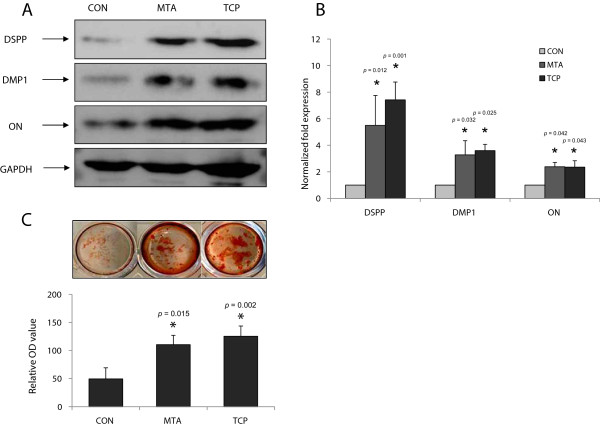
**Effects of the tested materials on odontoblastic differentiation of hDPCs. (A)** Effects of MTA and TCP on DSPP, DMP1, and ON protein in hDPCs. **(B)** The graph shows the quantification of protein expression by densitometry and is presented as fold increases compared with control cells. **(C)** Effects of MTA and TCP on the formation of calcification nodules in hDPCs. *Significant difference between each group; *P* < 0.05.

As shown in Figure 3A and B, the expression of DSPP, DMP1, and ON proteins in α-TCP- and MTA-treated cells was higher compared to the control group (*P* < 0.05). However, there was no significant difference between the two experimental groups (*P* > 0.05).

### Alizarin Red S staining

To investigate the effect of MTA and α-TCP on mineralization, hDPCs were stained with Alizarin Red S. The formation of mineralized nodules was significantly higher than it was in the medium only-treated cells of the control group at day 14 (*P* < 0.05). However, there was no significant difference between MTA and α-TCP treatments (*P* > 0.05) (Figure 3C).

### Immunofluorescence analysis

**Figure 4:**
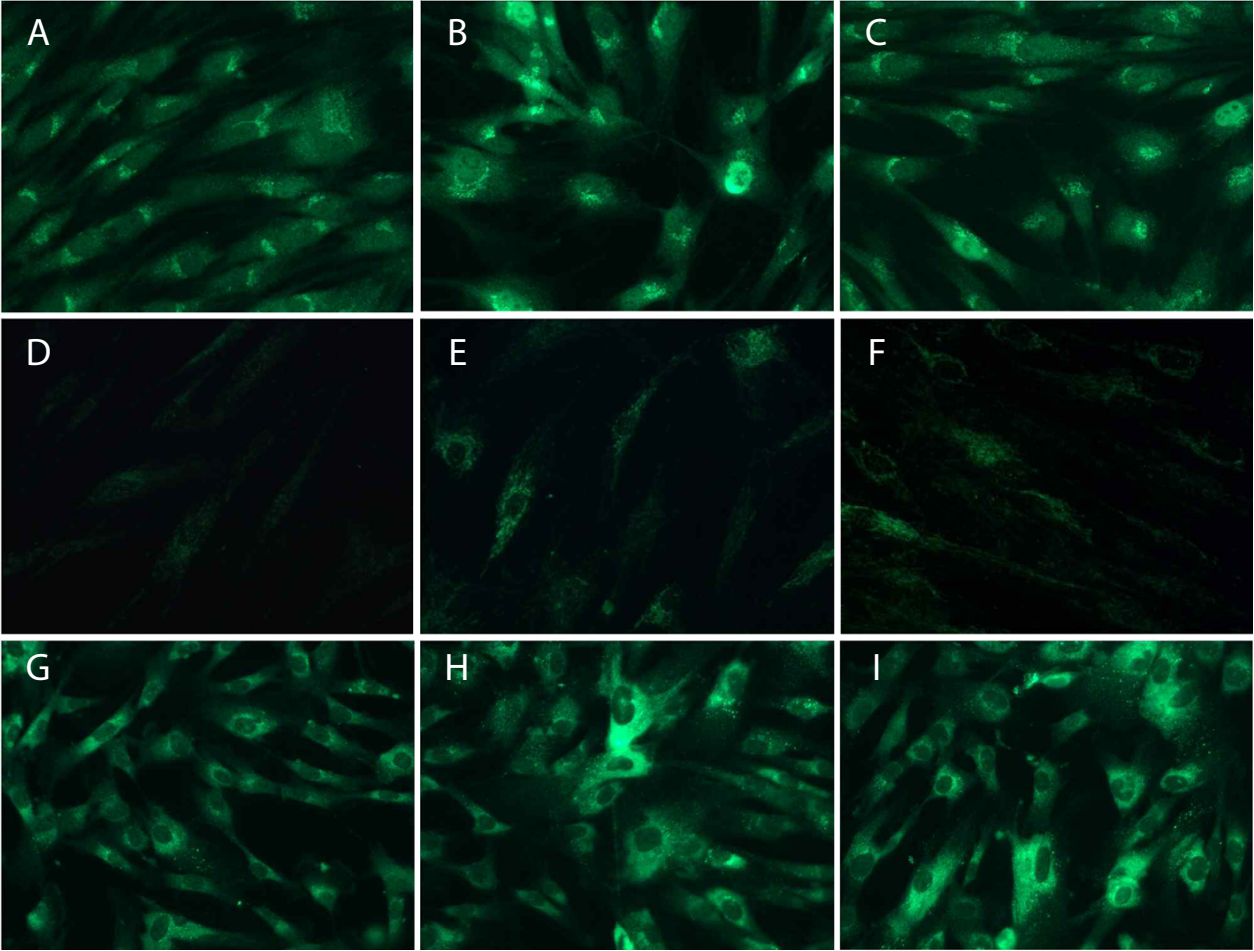
**Immunofluorescence analysis of hDPCs treated with medium only (A, D, and G), MTA (B, E, and H), or TCP (C, F, and I).** Fluorescence images showing anti-DSPP **(A-C)**, anti-DMP1 **(D-F)**, and anti-ON **(G-I)** signals (green) of cells after 3 days of culture (×400). Note that the protein signals in the cells of the experimental groups were stronger than those in the cells of the control group.

Immunofluorescence labeling was carried out to analyze the localization of the odontogenic-related proteins in hDPCs. DSPP, DMP1, and ON were localized in the cytoplasm, specifically in the perinuclear region of MTA- and α-TCP-treated cells. Furthermore, the protein signals in the cells of the experimental groups were stronger than those in the cells of the control group (Figure 4).

### Histological findings

**Figure 5:**
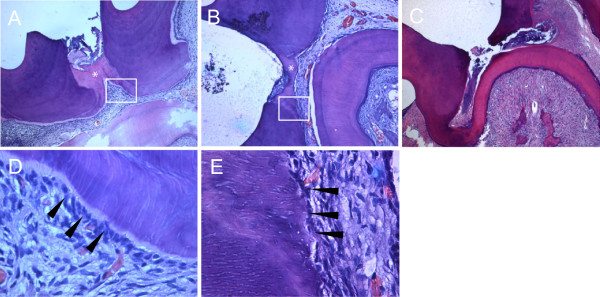
**Histological observation.** Capped pulps stained with hematoxylin-eosin 4 weeks after treatment with MTA **(A)** and TCP **(B)** (×50). **(C)** A specimen in the control group capped only with glass ionomer cement. **(D and E)** Higher magnification of boxed areas shown in A and B (×400), respectively. Odontoblasts (arrowheads) are polarized and appear to be arranged in a palisade pattern. *Reparative tertiary dentin formed underneath the capping materials.

Four weeks after treatment, tertiary dentin with complete continuity was formed directly underneath the capping material and the pulp exposure area in all samples of the two tested groups (Table 2). Notably, odontoblast-like cells were polarized and appeared to be arranged in a palisade pattern (Figure 5D and E). On the contrary, there was no tertiary dentin formation in the pulp exposure area of the control group (Figure 5C). There was no significant difference between α-TCP and MTA with respect to the continuity of tertiary dentin with either of the pulp capping materials (*P* > 0.05) (Table 2).

**Table 2:** Number of specimens attributed for each group of histological evaluation

	**Tertiary dentin formation**				
**Group**	**No. of specimen**	**1**	**2**	**3**	**4**
Control	4	0	0	0	4
MTA	6	6	0	0	0
TCP	6	6	0	0	0

There was no significant difference between MTA and TCP with respect to the formation of tertiary dentin with either of the pulp capping materials (*P* > 0.05).

## Discussion

The success of pulp capping is dependent on the preservation of vital pulp tissue and the formation of tertiary dentin [23,24]. For this purpose, MTA has been in widespread use clinically. However, MTA does not have good handling properties when prepared according to the manufacturers’ instructions, and the setting time is relatively long after mixing [20,25]. Meanwhile, CP cements have been interesting as a pulp capping agent, because of favorable biocompatibility and osteogenic/odontogenic potentials [13,26,27]. However, due to some drawbacks such as limited antibacterial properties, long setting time, and compressive strength, the application of CP cement for vital pulp therapy is limited [28,29]. Recently, fast-setting α-TCP cement was experimentally developed both for bone repair procedures and vital pulp therapy to overcome one of the physical disadvantages of conventional CP cements. Kurashina demonstrated that α-TCP-based cement is also a promising material as a bone substitute [1]. In fact, the β-type is considered a more popular TCP variant for bone repair, but the α-type offers more resistance to degradation by tissue [30,31]. This characteristic of α-type TCP would be more appropriate for vital pulp therapy. In fact, the present study is not the first study which attempted to show the potential clinical application of fast-setting CP cements. Miyamoto et al. reported that fast-setting CP cements may be used in a wide range of clinical fields, such as oral and maxillofacial surgery [32]. However, their study only investigated the setting behavior of the calcium phosphate cement, and did not investigate biological effects. In other words, there has been no study to determine whether the fast-setting α-TCP possesses odontogenic activity and can induce tertiary dentin formation, the ultimate goal of pulp capping. Therefore, we investigated its physical and biological/odontogenic effects in comparison with the currently used material, MTA.

First, we evaluated the physical properties of α-TCP including setting time, pH, solubility, and compressive strength in comparison with MTA. The setting time of α-TCP was significantly shorter than that of MTA (*P* < 0.05). α-TCP consists of small particles of CP. It is generally believed that the use of small particles increases the surface contact of the particles with the mixing liquid, which provides rapid setting and ease of handling. Due to this property, α-TCP might be used in a single-visit scenario without additional required appointment. On the contrary, the solubility of α-TCP was significantly higher than that of MTA (*P* < 0.05). This result was anticipated because CP cement, as a bone repair material, is essentially designed to be degraded and substituted by bone. However, this property can be considered negatively for pulp capping procedures. Furthermore, the compressive strength of α-TCP was significantly lower than that of MTA (*P* < 0.05). The ISO standard in terms of measuring compressive strength for a pulp capping material has not been developed. Therefore, ISO 3107:2004 was selected as a guideline for evaluation of the material properties. It is traditionally recommended that fillers be strong enough to resist the stress which is applied through an amalgam condensation [33]. Lately, however, tooth-colored, non-pressure-generated materials have been widely used instead of amalgam. In this respect, the importance of compressive strength is reduced for pulp capping materials.

Next, we investigated the biocompatibility of the two materials by evaluating the effects of the tested materials on cell morphology and viability. It is considered favorable for a pulp capping material to be biocompatible, because the material is then less likely to induce a response such as pulpal inflammation [34]. In our study, MTA and α-TCP had similar effects on cell viability shown by the MTT assay until day 7 (*P* > 0.05). On day 14, however, α-TCP showed higher cell viability compared to ProRoot (*P* < 0.05) (Figure 1D). Furthermore, SEM observations revealed that hDPCs cultured directly on MTA or α-TCP for 3 days appeared to be flat and exhibited well-defined cytoplasmic extensions (Figure 1C and D). Our study is supported by previous studies on the biocompatibility of CP cements [35,36], and indicates that the biocompatibility of fast-setting CP cement is comparable to that of MTA.

We also investigated whether α-TCP facilitated odontoblastic differentiation of hDPCs in comparison with MTA. MTA is considered to facilitate odontoblastic differentiation of hDPCs [37-40]. In addition, several studies have shown that CP cement has a similar mineralization ability compared to MTA [35,36,41]. In the present study, we showed that MTA and α-TCP promoted odontoblastic differentiation to a similar degree, as evidenced by the formation of mineralization nodules, and the expression of odontogenic-related markers. As shown in Figure 3, notably, the relative quantities of odontogenic-related markers such as DSPP, DMP1, and ON proteins were significantly higher in MTA- and α-TCP-treated cells compared to the medium only-treated cells of the control group (*P* < 0.05). However, there was no significant difference between the two experimental groups (*P* > 0.05). In immunofluorescence analyses, we observed that the signals in MTA- and α-TCP-treated cells were stronger compared to the cells of the control group (Figure 4). It is suggested that the ions released from MTA or CP cement, such as Ca, P, and Si, promote differentiation and mineralization of the cells through ion-mediated reactions [40,42,43]. EDS analysis of the current study revealed that MTA and α-TCP contain these elements, which might affect the expression of the mineralization phenotypes *in vitro* (Figure 2C and D). Overall, these results indicate that α-TCP possesses a similar ability with MTA in terms of promoting odontogenic potential of hDPCs.

Lastly, we investigated whether α-TCP induces the formation of tertiary dentin *in vivo*. Similar to *in vitro* results, there was no difference between MTA and α-TCP in terms of tertiary dentin formation (*P* > 0.05). Tertiary dentin with complete continuity was formed directly underneath the capping materials and the pulp exposure area in all samples of the two tested groups (Table 2 and Figure 5). There have been several *in vivo* studies indicating that CP-based cement induces the formation of tertiary dentin in direct contact with the material, and revealing its potential for use as a pulp capping agent [9,10,13,44]. The alkaline environment caused by a material in the pulp space is required for reparative dentin formation, because the alkalinity appears to result in mild stimulation of cell differentiation [45,46]. In the present study, both MTA and α-TCP had alkaline pH values that remained consistently high for 14 days, although the pH values of MTA were significantly higher than those of α-TCP (Figure 1A). Similar to our result, Tagaya et al. reported that the pH of the CP-based cement solution never exceed 8.0, even after about one month of storage, and this provides a mildly alkaline environment for pulpotomy [26]. Along with ionic release or other possible mechanisms, this alkaline environment might promote reparative dentin formation *in vivo*.

## Conclusions

The present study indicates that α-TCP has a similar odontogenicity to MTA both *in vitro* and *in vivo*, whereas it has a much quicker setting time. However, α-TCP showed inferior physical properties including solubility and compressive strength. Thus, the first null hyphothesis was rejected, but the second one was confirmed. Collectively, our results suggest that α-TCP is potentially suitable for use as an effective pulp capping material. However, long-term clinical evaluation is required with respect to the use of α-TCP.

## Abbreviations

DMP1: Dentin matrix protein 1; DMSO: Dimethyl sulfoxide; DSPP: Dentin sialophosphoprotein; EDTA: Ethylene diamine tetraacetic acid; FBS: Fetal bovine serum; GAPDH: Glyceraldehyde-3-phosphate dehydrogenase; hDPCs: Human dental pulp cells; IRB: Institutional Review Board; MEM: Minimal essential medium; MTA: Mineral trioxide aggregate; MTT: 3-(4,5-dimethylthiazol-2-yl)-2,5-diphenyltetrazolium bromide; ON: Osteonectin; PBS: Phosphate buffered saline solution; SDS-PAGE: Sodium dodecyl sulfate-polyacrylamide gel electrophoresis; SEM: Scanning electron microscopy; TCP: Tricalcium phosphate.

## Competing interests

The authors declare that they have no competing interests.

## Authors’ contributions

Min KS and Lee KW contributed to planning and designing the study, in the data analysis and submission of the manuscript. Lee JB performed most of the laboratory work. Park SJ and Kim HH performed the animal study. Kwon YS participated in the laboratory work and animal study. All authors have read and approved the final manuscript.

## Pre-publication history

The pre-publication history for this paper can be accessed here:

http://www.biomedcentral.com/1472-6831/14/87/prepub
